# Prediction of three-dimensional dose distribution for patient-specific quality assurance based on log files using WingsNet

**DOI:** 10.1186/s13014-025-02760-2

**Published:** 2025-11-20

**Authors:** Ying Huang, Yifei Pi, Ruxin Cai, Kui Ma, Hao Wang, Hua Chen, Hengle Gu, Yan Shao, Aihui Feng, Yanhua Duan, Zhenjiong Shen, Qing Kong, Zhiyong Xu, Weihai Zhuo

**Affiliations:** 1 Institute of Modern Physics, Fudan University, Shanghai, China; 2 Key Lab of Nucl. Phys. & Ion-Beam Appl. (MOE), Fudan University, Shanghai, 200433 China; 3 Shanghai Chest Hospital, Shanghai Jiao Tong University School of Medicine, Shanghai, 200030 China; 4 Department of Radiation Oncology, The First Affiliated Hospital of Zhengzhou University, Henan, China; 5 Varian Medical Systems, No.8 Yun Cheng Street, Beijing, China; 6 Institute of Radiation Medicine, Fudan University, Shanghai, 200032 China

**Keywords:** Three-dimensional dose distribution, Patient-specific quality assurance, Log files, Dose verification

## Abstract

**Background:**

This study aims to construct and train the WingsNet model, which leverages the parameters recorded in log files to rapidly and accurately predict the patient-specific three-dimensional (3D) dose distribution for IMRT quality assurance (QA).

**Methods:**

We conducted a retrospective analysis of data from 286 lung cancer patients treated with a prescription of 60 Gy in 30 fractions, with 242 cases used for model training and 44 for testing. Log files containing information such as multi-leaf collimator (MLC) positions, monitor units (MU), and gantry angles were collected from Varian treatment accelerators. Pylinac software was employed to extract mechanical parameters from the log files, generating 2D fluence maps, which were then converted into 3D volumes using a ray-tracing algorithm. CT images, RT structures, and 3D volumes were resampled to a uniform dimension of 128*128*128 to serve as input for the WingsNet model, with the 3D dose distribution calculated by the treatment planning system (TPS) serving as output. The model training utilized L1 loss and mean squared error (MSE) as evaluation metrics.

**Results:**

The results of this study demonstrate that the WingsNet model can effectively predict the 3D dose distribution of IMRT plans based on the parameters recorded in the log files. Evaluation through metrics such as mean absolute error (MAE), root mean square error (RMSE), and dose-volume histogram (DVH) indices reveals that the model performs well in most areas, with some errors observed in the planning target volume (PTV) region and at high dose levels, yet it retains potential for clinical use. Visually, the isodose line distributions are consistent. The Dice coefficients between the predicted and reference dose distributions at varying isodose line levels indicate a decreasing trend as the dose level increases.

**Conclusions:**

The WingsNet model developed in this study successfully predicts the patient-specific 3D dose distribution for QA by parsing the parameters recorded in log files. This model shows promise for use in 3D dose distribution verification for IMRT, providing an efficient and reliable tool for the verification of 3D dose distributions in patient-specific QA.

## Background

Intensity-modulated radiation therapy (IMRT) is a widely used radiotherapy technique in cancer treatment that delivers a higher dose to the target while reducing the dose to organs at risk (OAR), thereby providing a highly conformal dose distribution [1]. However, the complexity of IMRT often leads to increased uncertainty and variability in dose distribution [2], making the implementation of a reliable and robust quality assurance (QA) process crucial for ensuring dose accuracy and patient safety [3–5].

The most common QA methods in IMRT planning are pre-treatment patient-specific QA using both two-dimensional (2D) and three-dimensional (3D) methods, but the mainstream 2D dose verification techniques are limited by measurement dimensions and cannot fully assess the entire 3D dose distribution. To more accurately reflect the dose differences between the target and OAR, there is a need for dose-volume histogram (DVH) verification based on anatomical structures and 3D dose distribution verification. Traditional methods of 3D dose verification, such as measurement-based reconstruction using dosimeters and phantoms, and calculation-based algorithms [6–8], are resource-intensive and may be affected by the accuracy of third-party dose calculation engines, limiting their widespread application. The log-file method is considered an effective means to assess the accuracy of IMRT delivery [9–11], but it also faces limitations such as complex modeling, calibration during long-term use, relatively high cost, and longer computation time. Our team aims to establish a 3D dose distribution verification method based on this log file, hoping to overcome the aforementioned limitations as much as possible.

The rapid development of artificial intelligence (AI) in recent years has provided a new approach: using AI technology to improve the efficiency and accuracy of pre-treatment patient-specific QA. Studies have confirmed the potential of AI in predicting gamma passing rates (GPR) based on plan parameters or fluence [12–14], classification predictions [15, 16], and predicting dose difference trends and locations for plans that fail dose verification [17]. Our research team has conducted research on 2D and 3D GPR predictions based on log files, confirming the accuracy of log files for QA result prediction [18, 19]. Although AI technology has shown great potential in various aspects of QA, there has been no research specifically focused on the prediction of 3D dose distribution for patient-specific QA. This gap in the field presents an opportunity for exploration, aiming to achieve more precise and efficient 3D dose distribution prediction for patient-specific QA through AI technology.

In this study, we propose the WingsNet model, which utilizes log files generated during the accelerator delivery process to predict the 3D dose distribution for patient-specific QA. By converting recorded delivery parameters such as MLC positions, gantry angles, and monitor units (MUs) into 2D fluence maps and then transforming them into 3D volumes using the ray-traversal principle, the 3D dose distributions calculated by the treatment planning system (TPS) are used as the model’s output to train the 3D dose distribution prediction model. Using the actual parameters from log files, the predicted 3D dose distribution of the plan delivery parameters is used for the verification of patient-specific QA 3D dose distribution. We expect this work to enhance the accuracy and efficiency of 3D dose verification and to lay the foundation for the development of intelligent QA methods for 3D dose distribution verification.

## Methods

### Patients data

We retrospectively collected 286 lung cancer treatment plans from September 2022 to April 2024, with all plans prescribed at 60 Gy in 30 fractions. Every treatment plan consists of a series of CT scan, PTV contour, OAR contours, prescription dose, log files, and clinically delivered dose distribution calculated on Philips Pinnacle 9.10 TPS (Philips Healthy Fitchburg, WI, USA) with step-and-shoot. All plans satisfy the NCCN [20] guideline. They are normalized to ensure that 95% of PTV received 100% of the prescription dose. All plans were delivered on a Varian accelerator equipped with a Varian high-definition 120 MLC which consists of two banks of 60 MLC leaves, with the outer 28 leaves and inner 32 leaves on each side having widths of 0.5 and 0.25 cm, respectively.

### Process of 3D dose distribution prediction based on log files

**Fig. 1:**
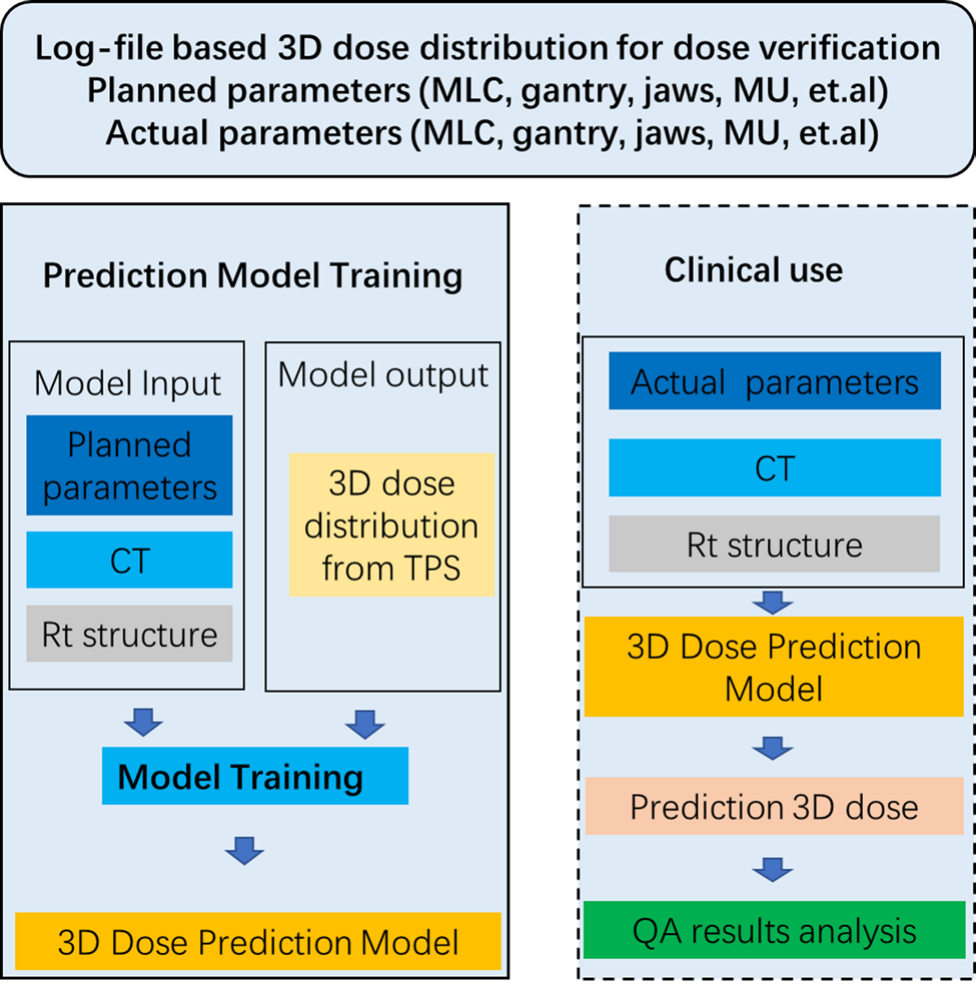
Scheme for patient-specific 3D dose distribution prediction based on log files

As shown in Fig. 1, the research on automated verification of 3D dose distribution based on log files includes model establishment and future clinical use. During the model establishment process, CT, PTV & OAR (left lung, right lung, total lungs, heart, spinal cord) masks, and a 3D volume generated from planned parameters recorded in the log files are used as input, with the 3D dose distribution calculated by the TPS serving as the output response for the prediction model. In the future clinical use process, patient CT scans, PTV & OAR masks, a 3D volume generated from actual parameters recorded in the log files are used as the input for the prediction model, which outputs the 3D dose distribution under the actual delivery parameters for the patient.

### Data preprocessing

**Fig. 2:**
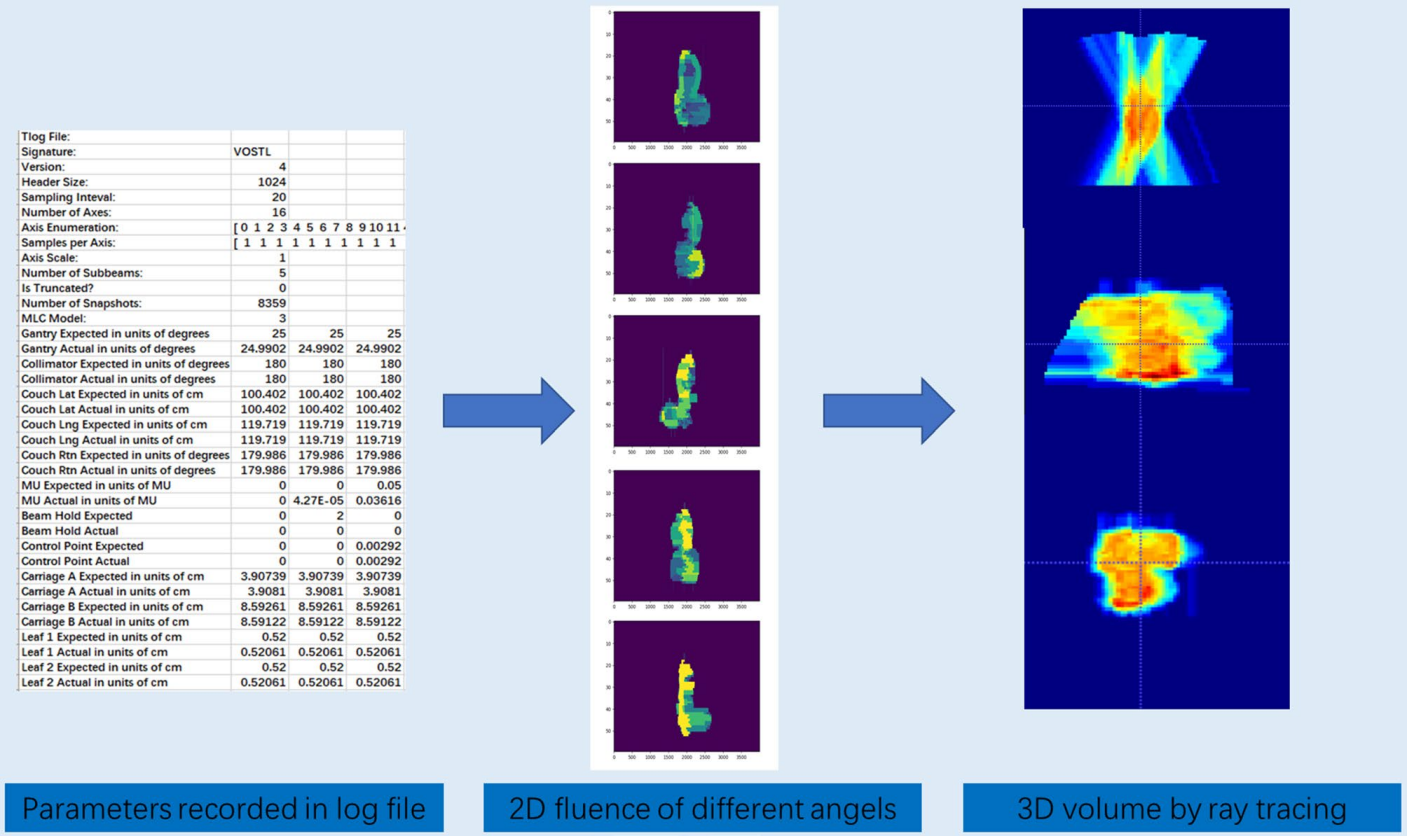
Flowchart of Converting Log File Parameters to 3D Volume. The process involves three main steps: (1) Raw machine parameters (e.g., MLC positions, MU) are extracted from the log files using Pylinac software (2). These 1D parameters are then converted into 2D fluence maps (3. A ray-tracing algorithm, specifically the three-dimensional digital differential analyzer (3D-DDA), is used to project the 2D fluence maps through the patient’s CT anatomy to generate a 3D dose volume, which serves as a key input for the WingsNet model

Due to the different dimensions of network input and output, this poses a challenge for network modeling. First, we utilized the Pylinac to parse the dynamic log files recorded by the Varian linear accelerator. These files document key machine parameters during treatment delivery at a high temporal resolution, primarily including the precise positions of the MLC at each recorded point, the cumulative MU, and the corresponding gantry angle. The raw one-dimensional (1D) time-series parameters cannot be directly used as input. Therefore, we converted them into a series of two-dimensional (2D) fluence maps. Specifically, for each recorded control point, we defined the field aperture using the MLC leaf positions at that moment and assigned the corresponding MU value as the intensity weight for that fluence map. This process yielded a set of 2D fluence maps described from the beam’s eye view (BEV) and synchronized with the gantry angles. Inspired by the work of Fan et al. on dose distribution prediction [21], an in-house developed algorithm based on the voxel traversal method was implemented to convert the 2D fluence map into a 3D volume. A widely used ray traversal algorithm is the three-dimensional digital differential analyzer (3D-DDA) algorithm [22]. This algorithm simulates the physical process of rays traveling from the radiation source, through the field aperture, and into the patient’s body. For each pixel on a 2D fluence map, the algorithm casts a ray from a virtual source point that traverses the patient’s CT voxel space. Each voxel along the ray’s path is assigned a value derived from the corresponding 2D fluence map pixel, attenuated based on distance according to the inverse square law. By processing all 2D fluence maps across all gantry angles, we ultimately generated a composite 3D volume that spatially encodes the dose delivery information described by all log file parameters. Figure 2 illustrates the conversion of log file parameters into a 3D volume. Finally, we registered 3D volume, generated from log file parameters, with the patient’s CT image and the 3D masks of the PTV and OARs. All three modalities of data were then uniformly resampled to a dimension of 128 × 128 × 128. These three data modalities together constitute the final input for the WingsNet model.

### Model introduction

In 3D dose distribution prediction, we face the challenge of uneven distribution of voxels at different dose levels, and the stacking of convolutional layers in traditional deep supervision methods often leads to gradient vanishing and explosion. Simply reducing the depth of the network is not a viable solution, as it may decrease the model’s representational power. To address this issue, we adopt a novel supervision method called group supervision and designed the corresponding network architecture – WingsNet [23], With group supervision, each group makes its own predictions based on a feature pyramid. In this way, each group can obtain information from all ConvBlocks within the group from different perspectives. Additionally, we establish a separate loss function for each group to ensure effective training for each group.

**Fig. 3:**
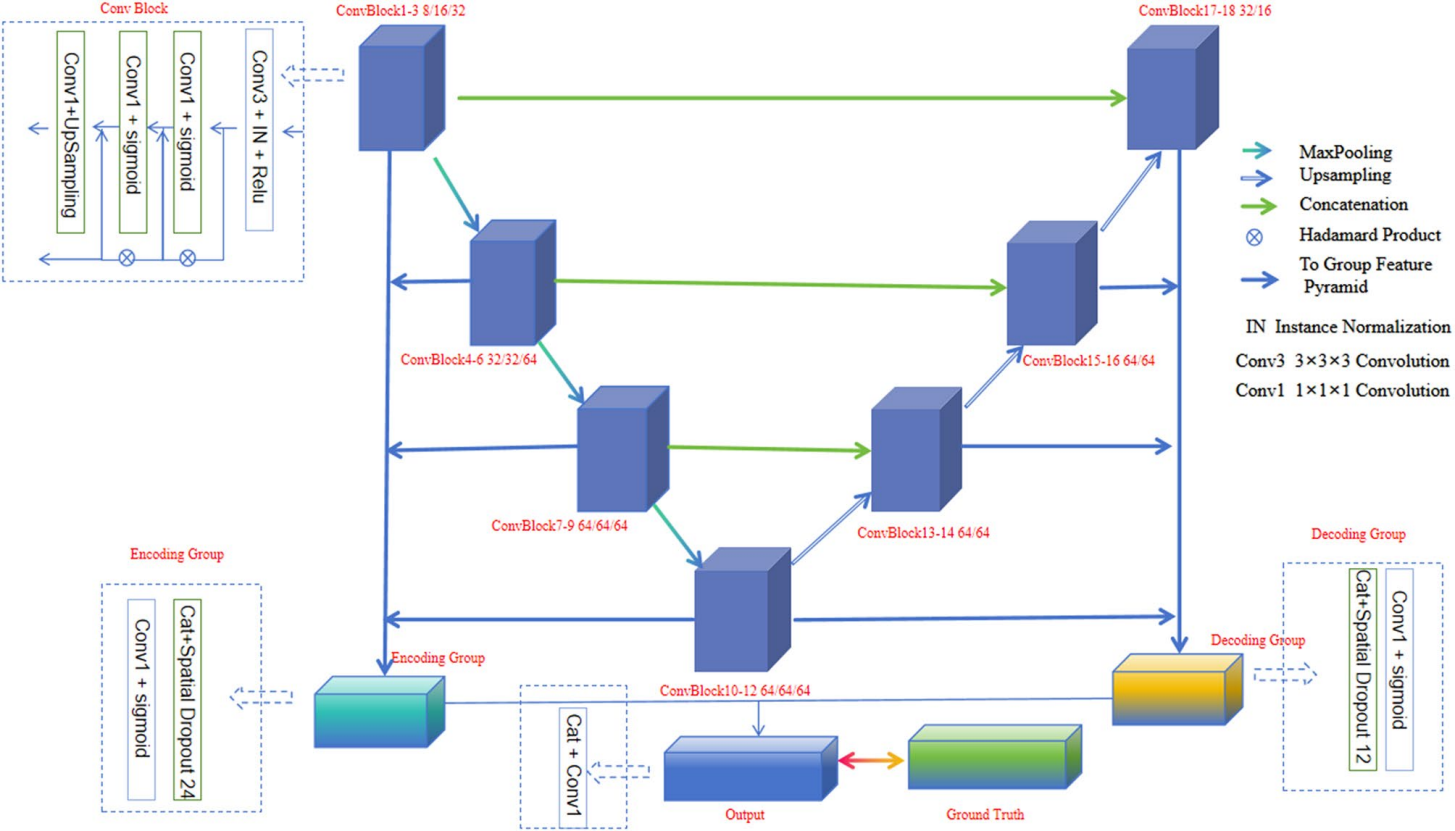
Architecture of WingsNet used for 3D dose distribution prediction

The constructed WingsNet is shown in the figure below (Fig. 3), consisting of 18 ConvBlocks, divided into an encoding group (ConvBlock 1–12) and a decoding group (ConvBlock 13–18). Each ConvBlock is composed of convolutional layers along with their corresponding normalization and activation layers. Within each block, in addition to the output path to the next block, there is an extra path to the group feature pyramid, which is a concatenation of the additional outputs of its member blocks. Specifically, the output of a ConvBlock is further processed through a 1*1*1 convolutional layer, and its size is restored to the original through an upsampling layer. The 1*1*1 convolutional layer can achieve a linear combination of features from multiple feature layers, facilitating cross-channel information integration. This is beneficial for feature extraction and information fusion to predict the dose for OAR, PTV, and 3D volume. Through this method of group supervision, we can effectively address the issues of gradient vanishing and explosion while maintaining the model’s representational power.

### Model training

We randomly selected 242 datasets for model training and 44 for model testing. Adam [24] was used as the optimizer with a learning rate of 6e-4 and a mini-batch size of 1 image. The experiments were conducted on a computer workstation with an Intel(R) Xeon(R) Gold 6338 CPU @ 2.00 GHz with 64 GB of main memory and a CUDA library on a graphics processing unit (NVIDIA A100 with 80 GB of memory). Network training of the WingsNet took approximately 24 h to run 200 epochs on the training and test datasets.

### Model evaluation

The evaluation metrics for the reference dose distribution and the predicted dose distribution include the Mean Absolute Error (MAE) and the Root Mean Squared Error (RMSE) of the voxels.

1 $${\text{MAE}} = \frac{1}{{vxl}}\sum {\mathop {vxl}\limits_{v = 1} } \left| {Dos{e_{predicted}}\left( v \right) - Dos{e_{ref}}\left( v \right)} \right|$$

2 $${\text{RMSE}} = \sqrt {\frac{1}{{vxl}}\sum {\mathop {vxl}\limits_{v = 1} } {{\left( {Dos{e_{predicted}}\left( v \right) - Dos{e_{ref}}\left( v \right)} \right)}^2}}$$

where,$\:{Dose}_{predicted}$ denotes the dose distribution map generated by WingsNet, $\:{Dose}_{ref}$represents the reference dose distribution map, and $\:{vxl}^{\:}$and $\:{v}^{\:}$denote the total number of voxels and the voxel index, respectively.

### Clinical results evaluation of the model

The evaluation of the predicted dose distribution against the reference dose distribution includes the dose differences for PTV and OARs. For PTV, we evaluate D_95_, D_98_, D_99_, D_max_, and D_mean_. D_x_ (Gy) indicates the dose covering PTV by x%. D_max_ and D_mean_ refer to the maximum and mean dose of the PTV, respectively. For total lungs, we assess V_5_, V_20_, and mean lung dose (MLD); for left and right lungs, we evaluate V_5_, V_20,_ and D_mean_; for the spinal cord, we evaluate D_max_; and for the heart, we evaluate V_30_, V_40_, and mean heart dose (MHD). Vx represents the percentage of the volume of the respective OAR receiving at least x Gy of dose. The similarity between the reference dose distribution and the predicted dose distribution is measured by the Dice similarity coefficient (DSC), defined as follows:

3 $$\:DSC=2\times\:\frac{{V}_{k}^{PD}\cap\:{V}_{k}^{R}}{{V}_{k}^{PD}+{V}_{k}^{R}}$$

where,

4 $$\:k=\frac{h}{{D}_{P}}\times\:100\%,\:h=0\dots\:{D}_{P},$$

$\:h$ is the threshold of dose to cut off a region of isodose volumes, $\:{V}_{k}^{C}$ and $\:{V}_{k}^{PD}$ is the isodose volume cut off by $\:k$ on clinical and predicted dose distribution in PTV.

## Results

### Comparison of reference and predicted dose distribution

**Fig. 4:**
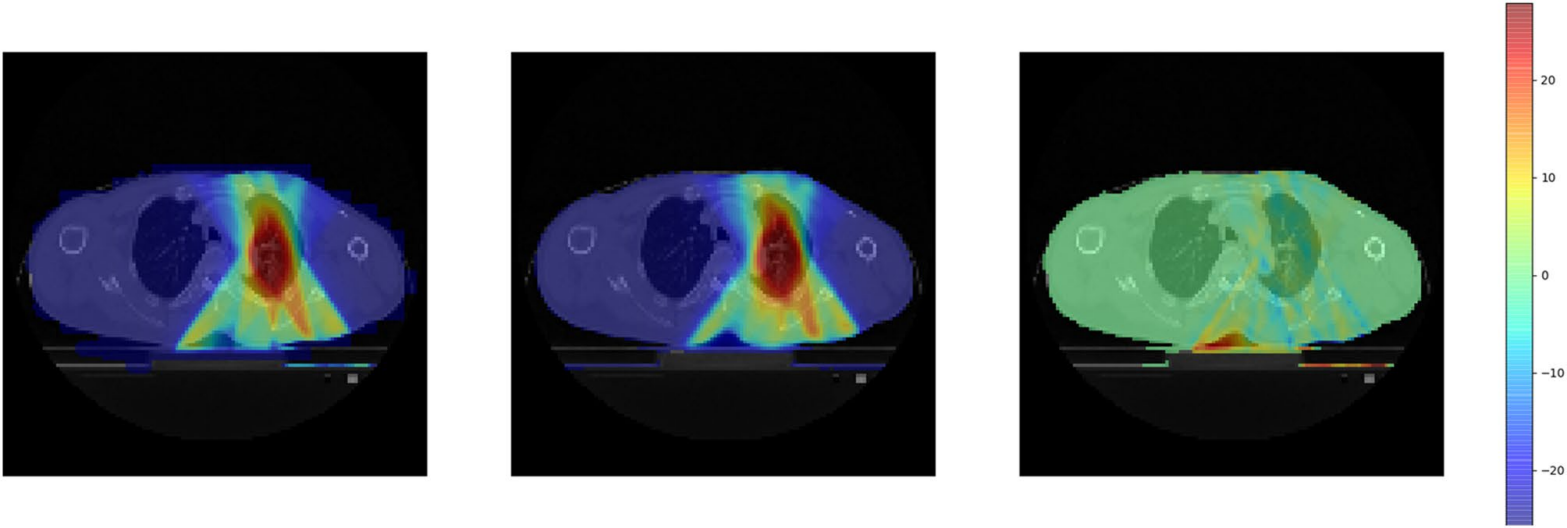
Dose distributions comparison in two axial planes for one patient: Reference (left), DL predicted (middle) and pixel-wise differences (right)

**Fig. 5:**
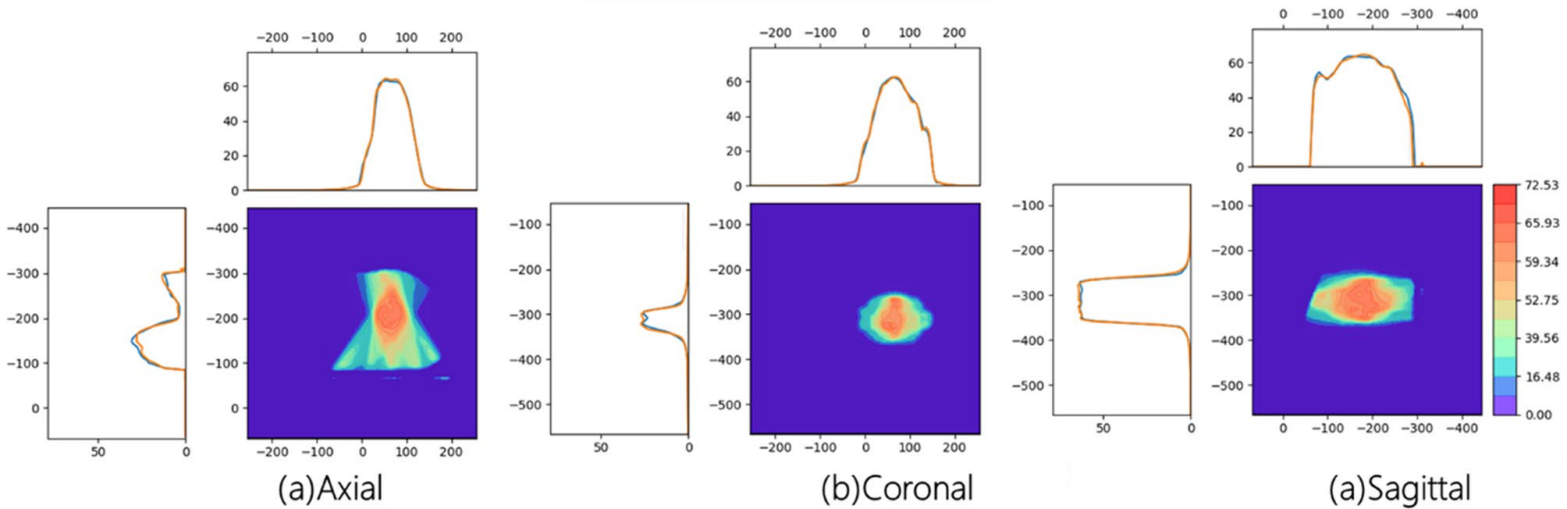
Dose profiles in different planes (Note: The blue line indicates the dose profile for the reference dose distribution, and the orange line represents the dose profile for the predicted dose distribution.)

Figure 4 displays the predicted dose distribution and the reference 3D dose distribution for a randomly selected patient, along with the dose difference relative to the reference dose distribution. The predicted 3D dose distribution closely aligns with the reference 3D dose distribution, indicating that WingsNet successfully predicts the 3D dose distribution based on parameters recorded in log files. The areas with relatively large dose differences are located at the edges of the body, which is a result of setting the learning range to the body during model training. Figure 5 presents the profile curves of the reference dose distribution and the predicted dose distribution. The two curves closely match across most regions, suggesting that our model can accurately predict the dose distribution. In the coronal-section, within the dose peak area (approximately 80% of the prescribed dose), the deviation between the predicted curve and the reference curve is less than 2%, indicating high prediction accuracy in critical treatment areas. However, it is important to note that the model may exhibit significant numerical deviations in high-gradient voxel regions where the dose changes most rapidly. Despite the strong agreement between the two curves, there is a slight deviation observed in the predicted curve at certain positions. This deviation might be due to the limitations of the model in dealing with complex tissue structures.

**Fig. 6:**
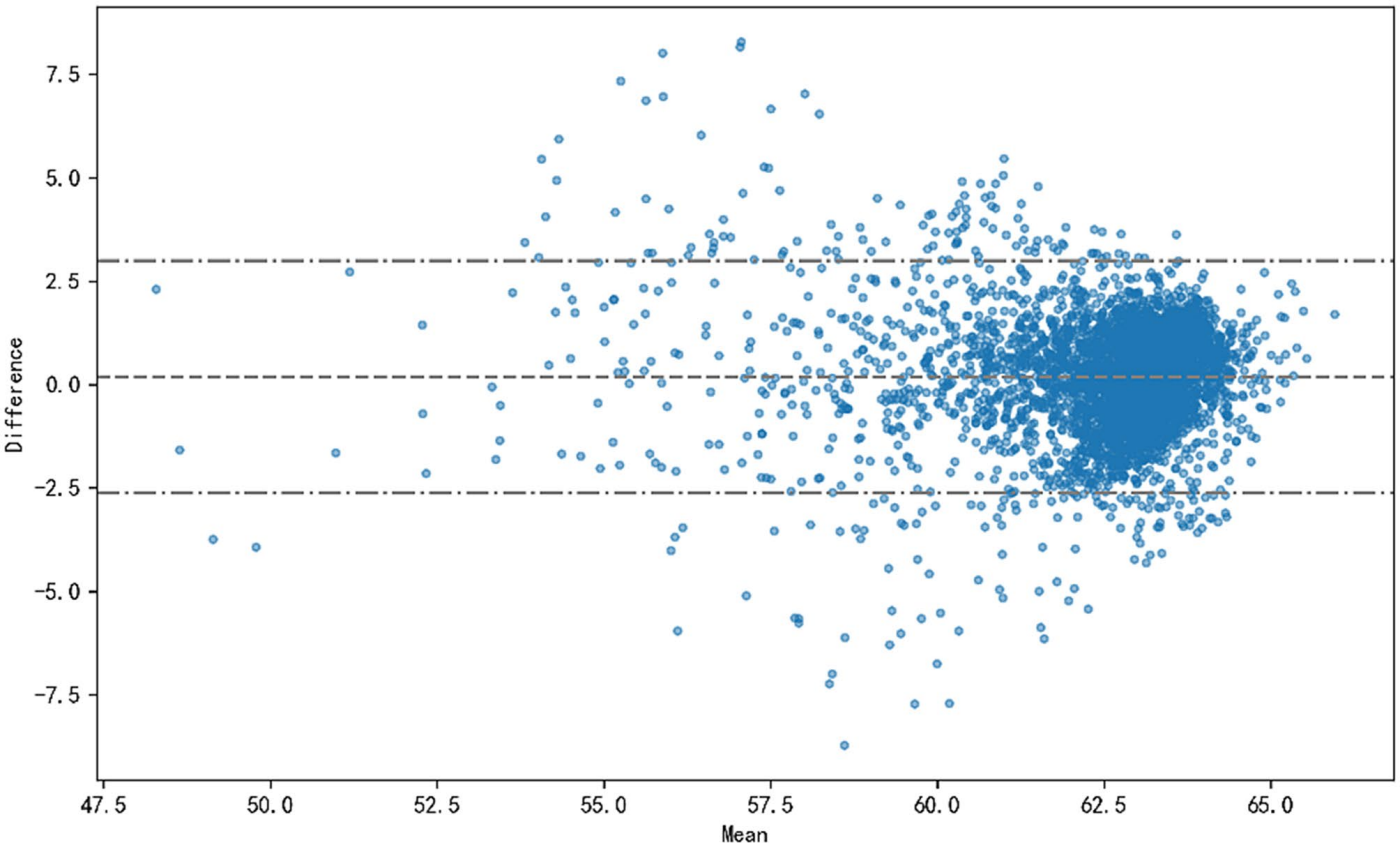
Bland and Altman analysis for PTV

### Error analysis in various regions of interest (ROI)

This study evaluates the accuracy of the radiotherapy dose prediction model in different ROI by calculating the MAE and RMSE (Table 1). The results show that across the entire dose distribution range, the model performs well with a MAE of 0.39 ± 0.12 and an RMSE of 8.49E-5 ± 3.91E-5, indicating a small deviation between the predicted dose distribution and the actual values. However, within the PTV, the MAE and RMSE are 1.27 ± 0.15 and 1.70 ± 0.22, respectively, showing relatively larger errors due to the complexity of the dose distribution in the PTV area.

**Table 1:** MAE and RMSE for different ROI regions

	MAE	RMSE
All	0.39 ± 0.12	8.49E-5 ± 3.91E-5
PTV	1.27 ± 0.15	1.70 ± 0.22
Spinal Cord	0.72 ± 0.27	1.49 ± 0.42
Heart	0.85 ± 0.46	1.56 ± 0.70
Left Lung	0.63 ± 0.47	1.16 ± 0.63
Right Lung	0.74 ± 0.44	1.33 ± 0.60
Total Lung	0.65 ± 0.20	1.32 ± 0.31

To further illustrate the differences between the predicted and reference dose distributions in the PTV, we used the Bland-Altman method to depict the agreement between the two dose distributions. Figure 6 shows the PTV dose errors and the 95% confidence intervals for a randomly selected patient. The horizontal axis of the graph represents the mean values, and the vertical axis represents the differences. In the middle of the graph, there are three horizontal lines, representing the upper limit of the 95% agreement limits, the zero limit, and the lower limit of the 95% agreement limits, respectively. The blue points in the graph represent the individual data points. According to the legend, we can see that most of the points fall within the 95% agreement limits, indicating that the predicted dose distribution has a good agreement with the reference dose distribution.

### DVH statistics of evaluation metrics

Table 2 analyzes the numerical values of the PTV and OAR dose metrics for the reference and predicted dose distributions, including the mean and standard deviation of the dosimetric indices of clinical interest in the reference and predicted dose distributions. Apart from the PTV metrics and the left lung (V_20_ and D_mean_), there is no statistically significant difference between the DVH parameters of the predicted dose distribution and those of the reference dose distribution. Although there are statistically significant differences in the PTV metrics between the reference and predicted dose distributions, the magnitude of these differences is small.

**Fig. 7:**
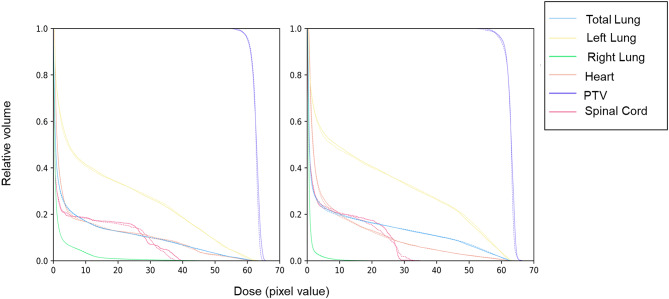
Comparison between the TPS calculated (solid line) and DL calculated (dashed line) OARs and PTV

Figure 7 shows the DVH curves for the PTV and OAR of two patients in the test set for both the reference and predicted dose distributions. As can be seen from the figure, except for a difference in the maximum value for the spinal cord, the DVH curves for the predicted dose distribution of different organs are very close to their corresponding reference DVH curves, indicating that our model can accurately predict the dose distribution within the patient’s body.

**Table 2:** The average value and standard deviation (mean ± SD) of relevant clinical indices for all the testing patients

Structure	Clinical indices	Predicted value	Reference value	*P* value	Mean relative deviation
PTV	D_95_	57.997 ± 1.079	58.593 ± 0.938	**0.000**	0.007 ± 0.005
	D_98_	55.153 ± 1.843	56.074 ± 1.965	**0.000**	0.024 ± 0.016
	D_99_	53.124 ± 2.327	54.095 ± 2.503	**0.000**	0.029 ± 0.020
	D_max_	65.704 ± 0.611	67.021 ± 1.212	**0.000**	0.022 ± 0.014
	D_mean_	62.254 ± 0.281	62.418 ± 0.400	**0.014**	0.006 ± 0.005
Total Lung	V_5_	0.355 ± 0.106	0.354 ± 0.103	0.616	0.019 ± 0.013
	V_20_	0.189 ± 0.050	0.189 ± 0.048	0.692	0.018 ± 0.011
	MLD	10.503 ± 2.666	10.519 ± 2.606	0.522	0.013 ± 0.011
Right Lung	V_5_	0.403 ± 0.240	0.401 ± 0.239	0.371	0.022 ± 0.020
	V_20_	0.225 ± 0.173	0.224 ± 0.171	0.229	0.052 ± 0.086
	D_mean_	12.385 ± 8.411	12.374 ± 8.348	0.794	0.019 ± 0.015
Left Lung	V_5_	0.345 ± 0.258	0.346 ± 0.260	0.554	0.039 ± 0.059
	V_20_	0.186 ± 0.197	0.188 ± 0.197	**0.044**	0.057 ± 0.081
	D_mean_	10.315 ± 9.516	10.377 ± 9.605	**0.036**	0.022 ± 0.019
Spinal Cord	D_max_	43.701 ± 6.812	43.625 ± 6.521	0.799	0.039 ± 0.021
Heart	V_30_	0.142 ± 0.091	0.146 ± 0.093	0.132	0.056 ± 0.074
	V_40_	0.097 ± 0.070	0.099 ± 0.070	0.074	0.068 ± 0.069
	MHD	11.615 ± 6.695	11.667 ± 6.715	0.457	0.030 ± 0.028

### Analysis of isodose comparison between reference and predicted dose distributions

**Fig. 8:**
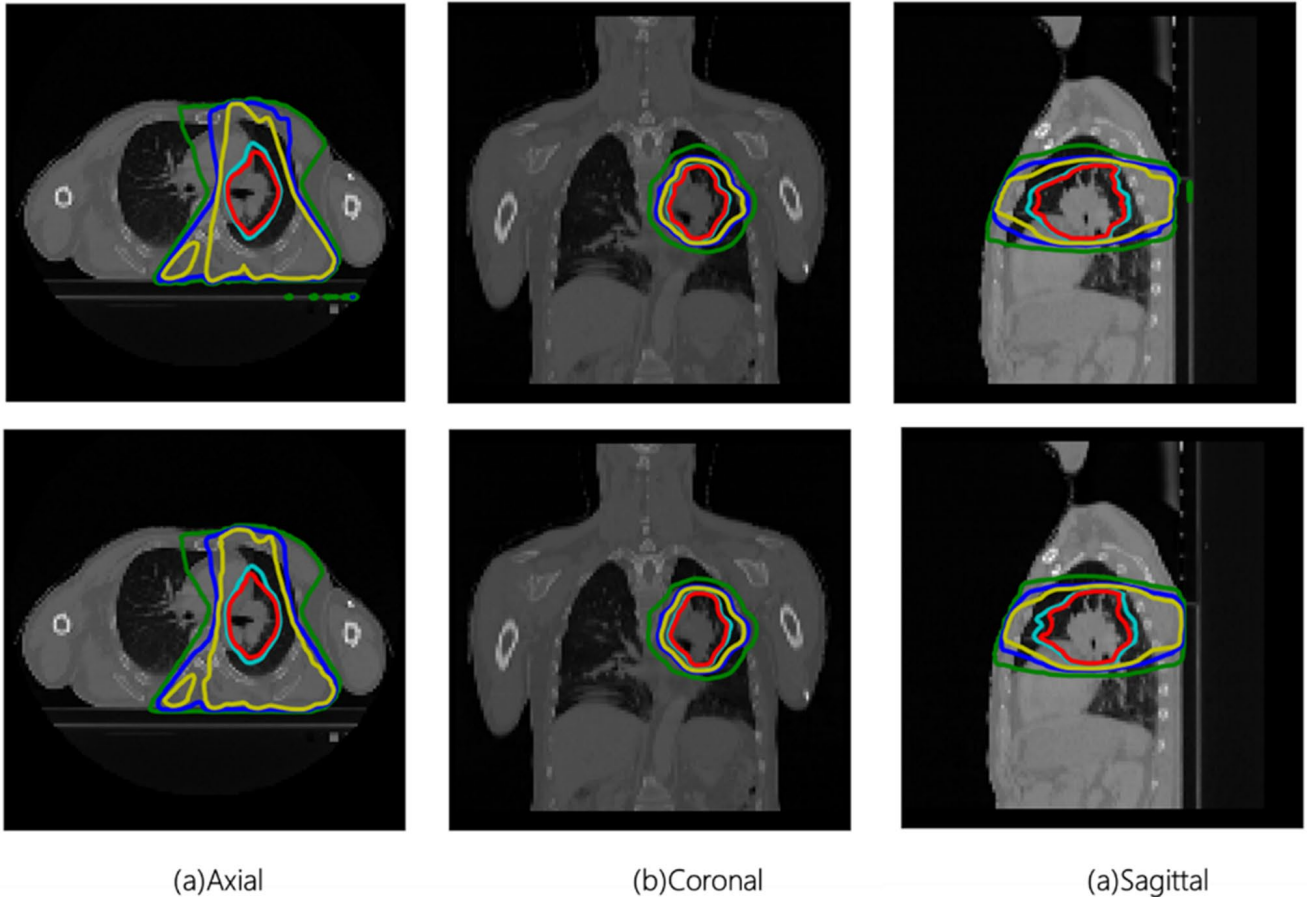
The examples of different dose levels for one patient

Figure 8 presents the results of the predicted dose distribution and the reference dose distribution for a patient at different isodose line levels. We observed that the isodose lines become increasingly concentrated and refined as the dose increases. In the low-dose region, the isodose line distribution is more extensive, covering a larger volume of tissue. Conversely, in the high-dose region, the isodose lines are more focused on the target, showing a higher dose gradient. From a visualization perspective, the consistency between different isodose distributions is relatively good.

**Fig. 9:**
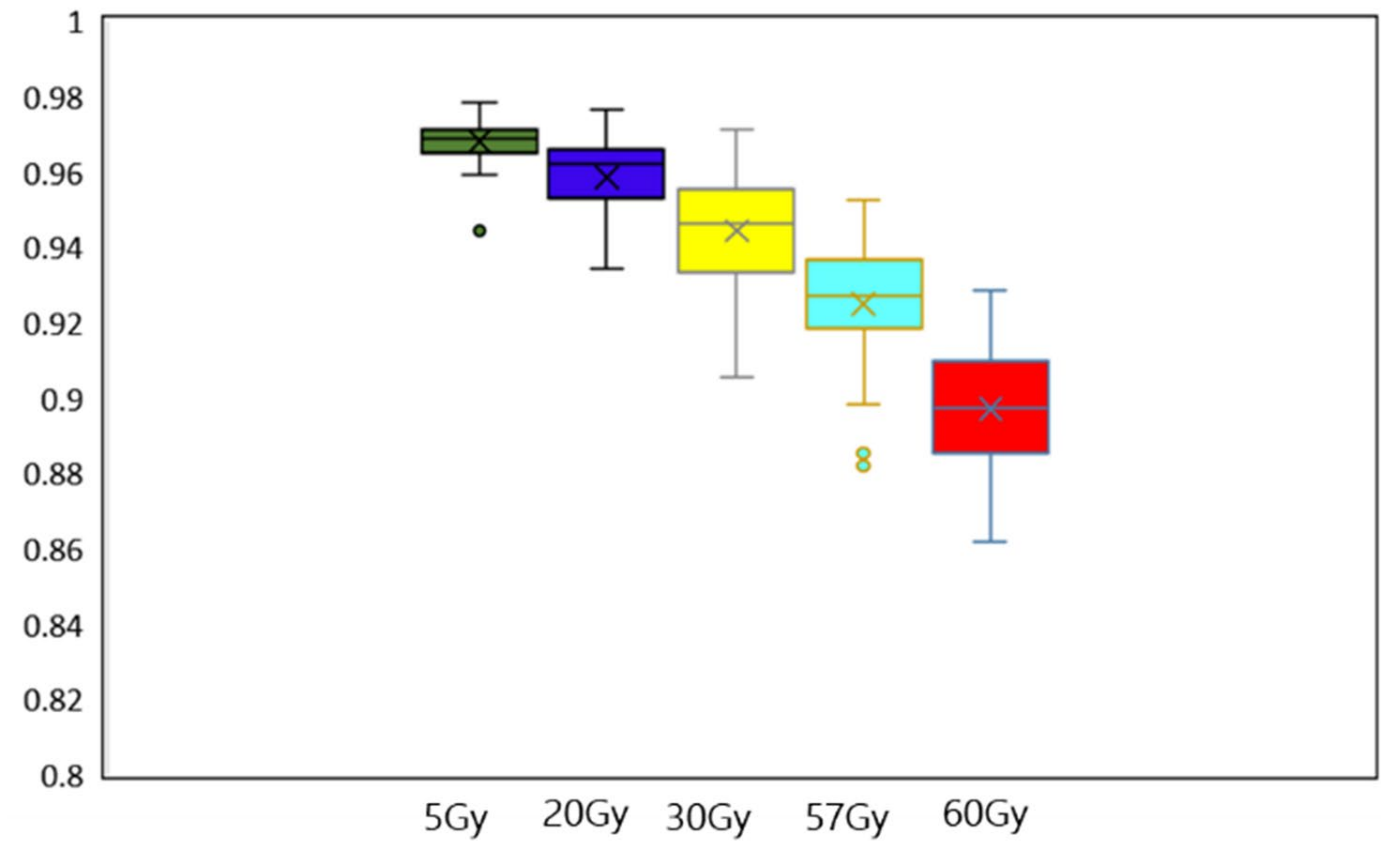
Comparing dice coefficients across different isodose volumes

We employed the DSC as a metric to assess the consistency of isodose lines between the reference and predicted dose distributions. The DSC ranges from 0 to 1, where 1 stands for a perfect match. The results showed that as the level of isodose lines increased, the Dice coefficient between the reference and predicted dose distributions gradually decreased (Fig. 9). Specifically, at lower dose levels (such as 5 Gy and 20 Gy), the Dice coefficients are higher, around 0.96 and 0.95, respectively, indicating a high degree of consistency of isodose between the reference and predicted dose distributions. However, as the dose level increases (such as 57 Gy and 60 Gy), the Dice coefficients significantly drop to around 0.92 and 0.90, respectively, indicating a noticeable decrease in consistency between the predicted and actual isdose lines at high isodose levels. This dose-dependent variation in the Dice coefficient suggests that our prediction model performs well in low-dose regions but experiences a decline in accuracy in high-dose situations. This may reflect the complexity of tumor shape and size variations under high-dose conditions, or the issue of overfitting in the model learning process. Future work should focus on improving the model’s performance in high-dose regions to enhance its application value in clinical practice.

## Discussion

In this study, we developed a model named WingsNet, which aims to accurately predict IMRT dose distribution using log file parameters. The results show a high degree of consistency between our predictions and the reference dose distributions, indicating that WingsNet has significant potential for application for patient-specific 3D dose distribution in IMRT QA. To our knowledge, this is the first study to explore the use of log file parameters for the prediction of patient-specific 3D dose distributions. The preliminary results not only demonstrate the prospects of this model in actual patient-specific QA applications but also offer a new perspective for the precision treatment in the field of radiotherapy.

In recent years, deep learning technology has achieved remarkable success in the field of radiotherapy, particularly in the prediction of 3D dose distribution, which has become a research focus [25, 26]. This technology has greatly facilitated the design process of radiotherapy plans by predicting the dose distribution in radiotherapy plans. For instance, our research team has successfully predicted the 3D dose distribution for lung cancer [27] and esophageal cancer [28] based on CT and RTstructure. Although existing studies mainly focus on using deep learning to optimize the design of treatment plans, the central role of 3D dose distribution prediction in the design of radiotherapy plans cannot be overlooked, and it is also key to the automation of patient-specific QA processes. In the QA process, accurate verification of the three-dimensional dose distribution is crucial for ensuring the safety and effectiveness of radiotherapy plans. Currently, automated QA faces challenges in the prediction of 3D dose distribution, but with the application of deep learning technology, new research trends have emerged, such as gamma passing rates prediction [12–14], classification of QA results [15, 16], dose difference trends and locations prediction [17], and error classification and magnitude prediction [29, 30], all of which have significantly improved the efficiency and precision of QA. This study aims to address the development bottleneck of automated QA for 3D dose prediction. By using widely recognized log files and combining them with deep learning technology, we successfully constructed the WingsNet network for predicting the 3Ddose distribution in IMRT. Our model not only provides detailed dose information for target and OAR for patient-specific QA but also avoids the cost of purchasing expensive 3D dose verification tools for radiotherapy centers, as well as the complex process of modeling based on accelerator data. Through transfer learning, radiotherapy centers can rapidly build prediction models suitable for their center based on historical data, greatly improving time efficiency. The technology for automated prediction of 3D dose distribution significantly shortens the working time in the traditional dose verification process, especially in aspects such as phantom setup, effectively solving the time-consuming issue in the independent dose verification process.

Log files meticulously record the key parameters during the delivery of radiotherapy plans, including planned and actual MU, MLC positions, jaws, gantry, and other parameters. These details are considered vital tools for verifying the accuracy of radiotherapy delivery, and their effectiveness and practicality have been confirmed in multiple studies [31]. After completing the equipment’s commissioning measurements and machine calibration following standard QA procedures such as AAPM TG 142 [32], the high temporal and spatial resolution data provided by log files greatly facilitate patient-specific QA. Our team’s previous studies have successfully predicted 2D and 3D QA results using these high-resolution log file data, including gamma passing rates for different threshold criteria, and has achieved certain results [18, 19]. This study demonstrated the feasibility of predicting 3D dose distribution from log file parameters, with the accuracy being intrinsically linked to GPR. Our model learns the complex relationships between all delivery parameters and the final dose. Beyond the core parameters we used (MLC position, MU, gantry angle), log files contain other data known to affect GPR, such as leaf speed, control point intervals, and dose rate stability, whose fluctuations impact MLC accuracy [33, 34]. Our predictive method is a comprehensive assessment that considers the cumulative effects of all these parameters. It implicitly captures the complex, non-linear relationships between all physical parameters and the final dose, which is the foundation for GPR calculation.

In the adaptation of the WingsNet algorithm for our study, we focused on enhancing the network’s capability to capture both local and global features within images, as well as improving its adaptability across various dose regions. By incorporating skip connections and attention mechanisms into the existing WingsNet architecture, the model is better equipped to handle complex dose distributions, which significantly boosts the accuracy of predictions. To further refine resolution and feature fusion, we utilized depthwise separable convolutions and pyramid pooling techniques. These methods not only preserve high resolution but also effectively integrate multi-scale feature information to enhance the precision of predictions. Moreover, we applied data augmentation strategies within the WingsNet framework to bolster the model’s generalization across different datasets and environments. Through these enhancements to the WingsNet architecture, we have successfully developed a more accurate model for predicting 3D dose distributions. Although this study demonstrated the effectiveness of our enhanced WingsNet model, we acknowledge that a direct comparative analysis with other state-of-the-art deep learning architectures was not conducted. Our initial focus was on establishing a robust and feasible method for 3D dose prediction from log files. Future work will include systematic benchmarking studies against other established models to validate the performance advantages of the WingsNet architecture for this specific application.

In this study, the 3D dose distribution prediction model we established exhibited significant numerical deviations in high-gradient voxel regions, such as the PTV, as evidenced by relatively large MAE and RMSE values. From the DVH statistical results, there were statistically significant differences between the DVH parameters of the predicted dose distribution and the reference dose distribution, indicating that the model had relatively large errors compared to the reference dose distribution when predicting the dose distribution in the high-dose region of the PTV. This phenomenon may be due to the more complex dose distribution in the PTV area and the relatively small number of voxels in the high-dose region, which limits the model’s learning ability in this area. In response to this issue, we recognize the need to strengthen the model’s learning ability in the high-dose PTV area in subsequent model optimization work, to improve the prediction accuracy in this critical region. We plan to achieve this goal by improving the model architecture or introducing more advanced training strategies, thereby ensuring that the model could provide more precise dose predictions in clinical applications.

This study has several limitations. Firstly, it only included lung cancer cases. This choice was driven by the fact that lung cancer is the most commonly diagnosed cancer and a leading cause of cancer mortality, and our institution, as a specialized thoracic hospital, possesses a large volume of lung cancer case data, which facilitated our initial exploration into the feasibility of 3D dose distribution prediction from log files. While this limits the model’s generalizability in its current form, the complex anatomical structures in lung cancer cases provide an extremely challenging learning environment for the model. We selected this specific cancer type as a starting point to establish a methodological foundation. In future, we will validate and adapt the model to other anatomical sites, such as the head and neck and prostate, through multi-center collaborations and transfer learning strategies to enhance its generalizability and clinical utility. Secondly, we did not compare the model-predicted 3D dose distributions with the outputs from commercial 3D QA systems. We used the dose distribution calculated by the TPS as the reference standard, and our results demonstrated a high degree of agreement with the TPS dose, which successfully proves the fundamental feasibility of our method. Future work by our team will involve cross-validation with independent 3D dose reconstruction software to prepare for clinical translation. Furthermore, in future, we will perform a sensitivity analysis of our model. This will involve systematically introducing perturbations into the log file inputs—such as MLC leaf position errors, MU discrepancies, and gantry angle inaccuracies—to quantify the model’s sensitivity to these errors and to establish detection thresholds. A sensitivity analysis is crucial for validating whether the model can serve as a reliable, automated error detection tool. This will advance our method from nominal dose prediction towards a comprehensive QA solution.

In summary, our research provides preliminary evidence for the prediction of 3D dose distribution based on log files and opens a new path for the automation and precision of patient-specific QA in IMRT. We expect that future work will further refine the model and see widespread application in clinical practice.

## Conclusion

This study explored a method based on log files to predict 3D dose distribution. Our results indicate that the WingsNet model can accurately predict the 3D dose distribution for patients. This novel method of 3D dose distribution quality control offers the potential to enhance the efficiency and accuracy of patient-specific QA, and its application prospects in clinical practice are promising.

## Data Availability

The datasets generated during and/or analyzed during the current study are available from the corresponding author on reasonable request. The data are not publicly available due to participant privacy concerns. For further inquiries, please contact Zhiyong Xu at xzyong12vip@sina.com.

## Abbreviations

3D: Three-dimensional
QA: Quality assurance
MLC: Multi-leaf collimator
MU: Monitor units
TPS: Treatment planning system
MSE: Mean squared error
MAE: Mean absolute error
RMSE: Root mean square error
DVH: Dose-volume histogram
PTV: Planning target volume
IMRT: Intensity-modulated radiation therapy
OAR: Organs at risk
2D: Two-dimensional
AI: Artificial intelligence
GPR: Gamma passing rates
3D-DDA: Three-dimensional digital differential analyzer
MLD: Mean lung dose
MHD: Mean heart dose
DSC: DICE similarity coefficient
ROI: Regions of interest
